# Transmissible gastroenteritis virus targets Paneth cells to inhibit the self-renewal and differentiation of Lgr5 intestinal stem cells via Notch signaling

**DOI:** 10.1038/s41419-020-2233-6

**Published:** 2020-01-20

**Authors:** Aimin Wu, Bing Yu, Keying Zhang, Zhiwen Xu, De Wu, Jun He, Junqiu Luo, Yuheng Luo, Jie Yu, Ping Zheng, Lianqiang Che, Xiangbing Mao, Zhiqing Huang, Lan Wang, Jun Zhao, Daiwen Chen

**Affiliations:** 1 Institute of Animal Nutrition, Sichuan Agricultural University, Chengdu, China; 2 Key Laboratory for Animal Disease-resistance Nutrition of China Ministry of Education, Sichuan Agricultural University, Chengdu, China; 3 College of Veterinary Medicine, Sichuan Agricultural University, Chengdu, China

**Keywords:** Differentiation, Disease model, Self-renewal, Stem-cell differentiation, Stem-cell niche

## Abstract

Infection with transmissible gastroenteritis virus (TGEV) has been associated with villous atrophy within 48 h, which seriously disrupts intestinal homeostasis. However, the underlying mechanisms remain elusive. In this study, we found that TGEV infection severely disrupted intestinal homeostasis via inhibition of self-renewal and differentiation in Lgr5 intestinal stem cells (ISCs). Profoundly, TGEV-encoded NSP10/NSP16 protein complex-mediated the inactivation of Notch signaling provided a mechanistic explanation for this phenomenon. Initial invasions by TGEV-targeted Paneth cells through aminopeptidase N (APN) receptor, then inducing mitochondrial damage and ROS generation in them, ultimately causing Paneth cell decrease and loss of Notch factors (DII4 and Hes5), which are essential for Lgr5 ISCs self-renewal and differentiation. Interestingly, loss of Notch signaling induced goblet cells differentiation at the cost of absorptive enterocytes and promoted mucins secretion, which accelerated TGEV replication. Therefore, the more differentiation of goblet cells, the greater TGEV infection in jejunum. These results provide a detailed mechanistic pathway by which villous atrophy sharply occurs in TGEV-infected jejunum within 48 h. Thus, the pathogenesis of TGEV can be described as a “bottom up scenario”, which is contrary to the traditional “top down” hypothesis. Together, our findings provide a potential link between diarrheal virus infection and crypt cells response that regulates Paneth cells function and Lgr5 ISCs fate and could be exploited for therapeutic application.

## Introduction

The mammalian intestinal epithelium exhibits rapid self-renewal, with complete turnover of epithelial cell lining every 4–5 days^1,2^. In addition to its role in nutrient digestion and absorption, the intestinal epithelium also forms as a critical barrier against luminal pathogens to maintain intestinal homeostasis^3^. However, this barrier function is known to be susceptible to irradiation, chemical injury or infection with pathogens^4^. One of the central mechanisms to maintain intestinal barrier function is through activating crypt cells (Paneth cells and intestinal stem cells, ISCs)^5^. Whereas the mechanism by which irradiation or chemical toxins activate crypt cells response has been extensively studied^4,6^, far less is known about the response of crypt cells to diarrheal virus infection, such as TGEV.

TGEV, together with the human coronaviruses (HCoV-NL63 and HCoV-229e), belongs to the genus *Alphacoronavirus* within the subfamily *Coronaviridae*^7^, which are single-stranded, positive-sense RNA viruses relevant in animal and human health^8^. Generally, TGEV causes transmissible gastroenteritis with high morbidity in pigs of all ages and as high as 100% mortality in newborn piglets, especially those within 2 weeks of birth^9^. Most notably, TGEV not only causes devastating impact on the global pig industry, but also is a potential threat to human health, as its infection suppresses protein translation in diverse human cells^10,11^. The economic and health implications of TGEV have aroused significant concern worldwide^9^.

The typical symptomatic pathway of TGEV infection is villous atrophy within 48 h, followed by crypt hyperplasia, concomitant with lethal watery diarrhea, and ultimately severe dehydration in piglets until death^12^. TGEV infection causes intestinal barrier dysfunction and disrupts intestinal homeostasis^9,13^, which could affect intestinal epithelium renewal. The intestinal homeostasis and intestinal epithelium renewal are normally sustained by fast-cycling stem cells located around the base of the crypt^14^. These internal cycling stem cells are distinguished by Lgr5 expression, which is suggested to mediate cell proliferation in a number of tissues^15^. Small populations of Lgr5 ISCs regularly divide to produce highly proliferative progenitors known as transit-amplifying (TA) cells^14^ and terminally differentiate into the absorptive (enterocytes) or secretory (Paneth cells, goblet cells and enteroendocrine cells) cell lineages while gradually migrating upwards toward the top of the villi^16^. One exception to this migratory path is Paneth cells, which follow a downward migratory path to the crypt bottom and intermingle with Lgr5 ISCs^17^. The close interaction of Lgr5 ISCs and Paneth cells is essential for maintaining Lgr5 ISCs fate^18^, since Paneth cells provide crucial niche factors (such as Wnt3a, BMP, and Notch factors) for Lgr5 ISCs self-renewal and differentiation^19^. Of particular significance, Notch factors regulate TA differentiation status and intestinal developmental pattern^20^. Paneth cells express Notch ligands, which bind the Notch receptor on Lgr5 ISCs to activate expression of downstream genes, such as *Hes1* and *Hes5*^21,22^. These Notch target genes are essential for ISCs homeostasis, as inhibition of the Notch signaling results in complete conversion of epithelial cells into goblet cells both in vitro and in vivo^23,24^. In contrast, activation of the Notch signaling promotes proliferation to the absorptive cell lineage with a concomitant loss in secretory cell lineage differentiation^25^. Among Notch ligands, DII1 and DII4 are essential, and inhibition of both results in the loss of stem and progenitor cells^22^.

In this study, we explore how TGEV infection targets Paneth cells and through them disrupt Lgr5 ISCs and their ability to regenerate and differentiate, as well as adjacent effects on goblet cells and their role in maintaining the protective lining of the intestinal epithelium. We challenged the assumption that TGEV disruption of the jejunum occurs via a “top down” pathway with direct effects on the crypt cells, and instead consider how TGEV may function as a “bottom up” scenario with infection of Paneth cells initiating a cascade of events that ultimately inhibit intestinal epithelial homeostasis. These findings revealed a previously unrecognized link between diarrheal virus and crypt cells response that modulates ISCs homeostasis and could be exploited for therapeutic application.

## Results

### TGEV infection disrupts intestinal homeostasis

**Fig. 1:**
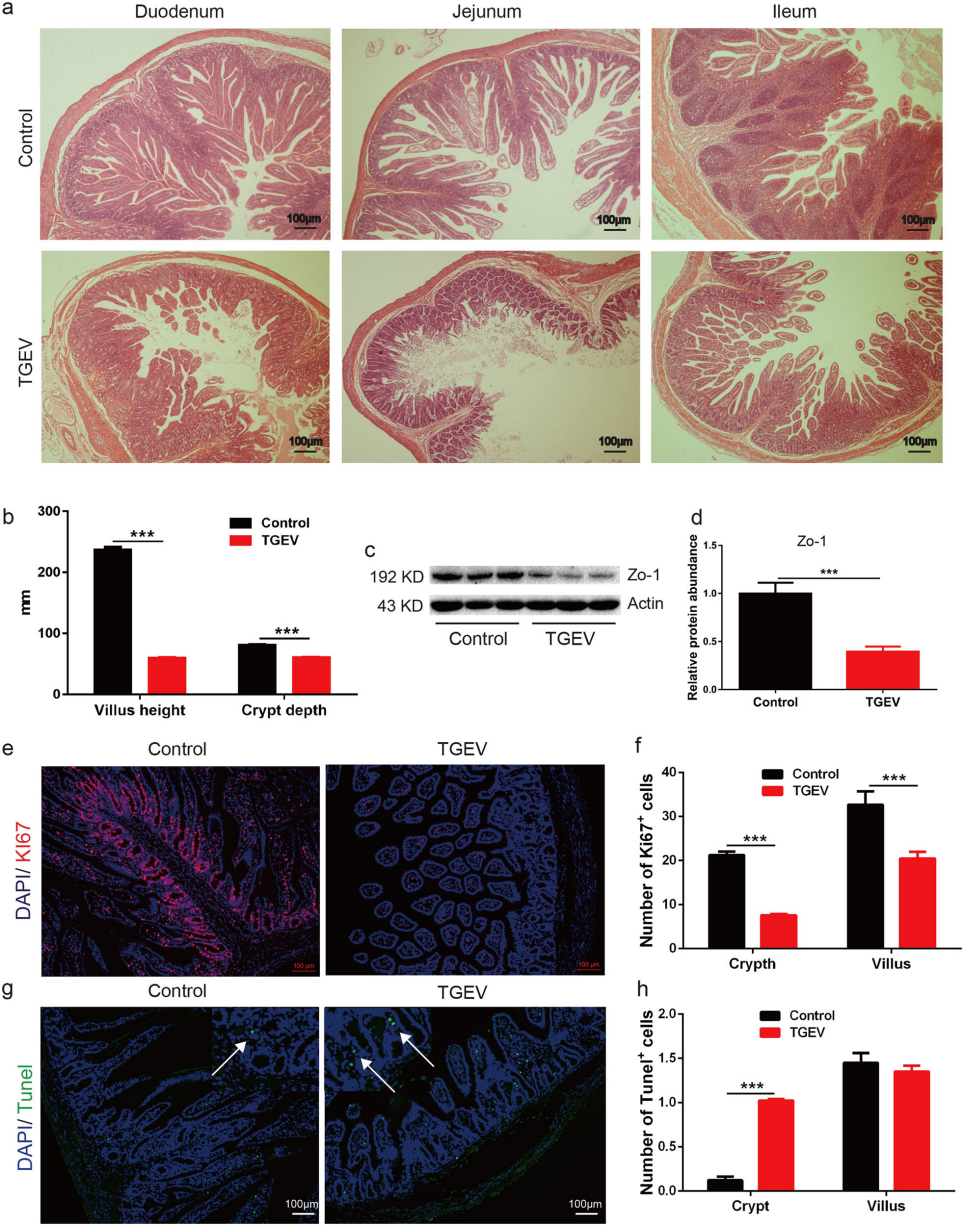
TGEV infection destroys the integrity of intestinal architecture and disrupts intestinal homeostasis. **a** Representative H&E stained cross-section showing villous atrophy and enterocytes shedding in TGEV-infected intestine. Scale bar, 100 μm. **b** Quantification of villous height (*n* = 150) and crypt depth (*n* = 150) in TGEV-infected jejunum (*n* = 3). **c** Western blot for junction protein Zo-1, Occludin, and Claudin-1 of jejunum from control and TGEV-infected piglets. Actin serves as a control. **d** Quantitation of bands to demonstrate the protein level of Zo-1. **e**, **f** Jejunal cross-section stained with KI67 (Scale bars, 100 μm) and quantification of the proliferation cells per crypt (*n* = 150) and villus (*n* = 150). **g** Cross-section of jejunum from control and TGEV-infected piglets stained with Tunel (the white arrows show cell apoptosis in crypt; Scale bars, 100 μm). **h** Quantification of necrotic cells per crypt (*n* = 150) and villus (*n* = 150).

As TGEV major targets organ is small intestine. Therefore, we collected the duodenum, jejunum and ileum tissues for intestinal architecture analysis. Histological analysis of these samples revealed that TGEV significantly disrupted intestinal morphology in small intestine, especially in jejunum (Fig. 1a). The most striking features were villous atrophy. Villous height decreased sharply, accompanying with enterocyte shedding, followed by crypt hyperplasia (Fig. 1a, b). Subsequently, the representative tight junction proteins Zo-1, Occludin, and Claudin-1 were determined by using western blot (WB). The results indicated that TGEV solely down-regulated Zo-1 protein level in infected jejunum (Fig. 1c, d). Moreover, we found that TGEV infection robustly halted cell proliferation both in crypt and villous (Fig. 1e, f), as assessed by Ki67 staining. Unlike cell proliferation, TGEV induced cell apoptosis solely in crypt cells, as detected by TUNEL staining (Fig. 1g, h). These results also were confirmed in IPEC-J2 cells (a jejunal epithelial cell line). TGEV infection not only inhibited cell proliferation, but also induced cell apoptosis in 36 h post infected cells (Supplementary Fig. S1). Together, these data strongly suggest that TGEV infection inhibits epithelial self-renewal and disrupts intestinal homeostasis.

### TGEV infection inhibits the self-renewal of Lgr5 ISCs and the differentiation of absorptive lineage

**Fig. 2:**
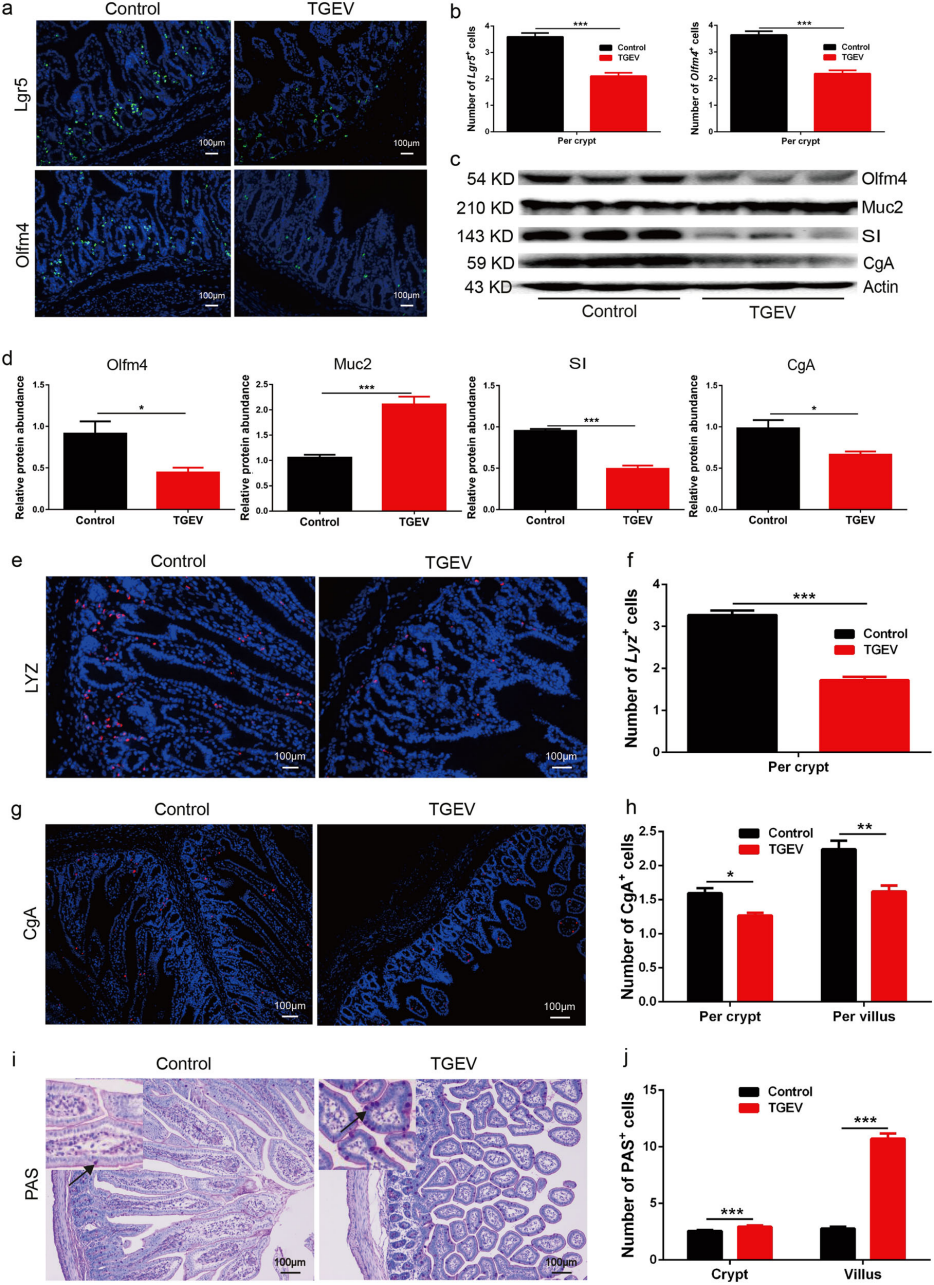
TGEV infection inhibits the self-renewal of Lgr5 ISCs and the differentiation of absorptive lineage. **a** Fluorescence in situ hybridization with a probe for *Lgr5* and *Olfm4* performed on the jejunum, revealing TGEV infection decreases the mRNA expression of *Lgr5* and *Olfm4*. **b** Quantification of Lgr5 and Olfm4 stem cells number per crypt (*n* = 150). **c** Western blot for junction protein Olfm4, Muc2, SI, and CgA of jejunum from control and TGEV-infected piglets. Actin serves as a control. **d** Quantitation of bands to demonstrate the protein level of Olfm4, Muc2, SI and CgA. **e**, **f** Fluorescence in situ hybridization with a probe for *Lyz* (Paneth cells) performed on the jejunum, and quantification of Paneth cells per crypt (*n* = 150). **g**, **h** Jejunal cross-section stained with CgA shows enteroendocrine, and quantification of enteroendocrine per crypt (*n* = 150) and villus (*n* = 150). **i**, **j** Representative jejunal PAS stained cross-section, showing goblet cells (black arrows) and quantification of goblet cells per crypt (*n* = 150) and villus (*n* = 150), (Scale bars, 100 μm).

To test the hypothesis that TGEV infection disruption intestinal homeostasis through inhibiting the self-renewal and differentiation of Lgr5 ISCs, WB, FACS, FISH and IF staining were used to determine the self-renewal of Lgr5 ISCs in TGEV-infected jejunum. We observed a significant decrease in Lgr5 ISCs number within crypt tissues for TGEV-infected jejunum (Fig. 2a, b). Similarly, Olfm4, another marker of ISCs, was repressed (Fig. 2a–d). In parallel, similar results were found in TGEV-infected IPEC-J2 cells (Supplementary Fig. S2a, b). We then examined the differentiation pattern of Lgr5 ISCs in TGEV-infected jejunum. The number of enterocytes was distinctly decreased in TGEV-infected jejunum, as revealed by SI protein expression (Fig. 2c, d). Similar results were determined in Paneth cells (Fig. 2e, f). Moreover, TGEV infection not only decreased the number of Paneth cells in crypts, but also caused enteroendocrine cells loss in crypts and villi (Fig. 2c, d, g, and h). In contrast, Muc2 protein level and PAS staining showed that TGEV infection strongly increased the number of goblet cells both in crypts and villi (Fig. 2c, d, i, and j). Subsequently, we found that TGEV infection not only halts cell proliferation and but also induces cell apoptosis in Lgr5 ISCs (Supplementary Fig. S2c, d). Together, TGEV infection induces goblet cells differentiation at the cost of absorptive enterocytes in TGEV-infected jejunum and severely alters Lgr5 ISCs fate.

### TGEV infection causes Paneth cells loss and disrupts the Notch signaling for Lgr5 ISCs self-renewal and differentiation

**Fig. 3:**
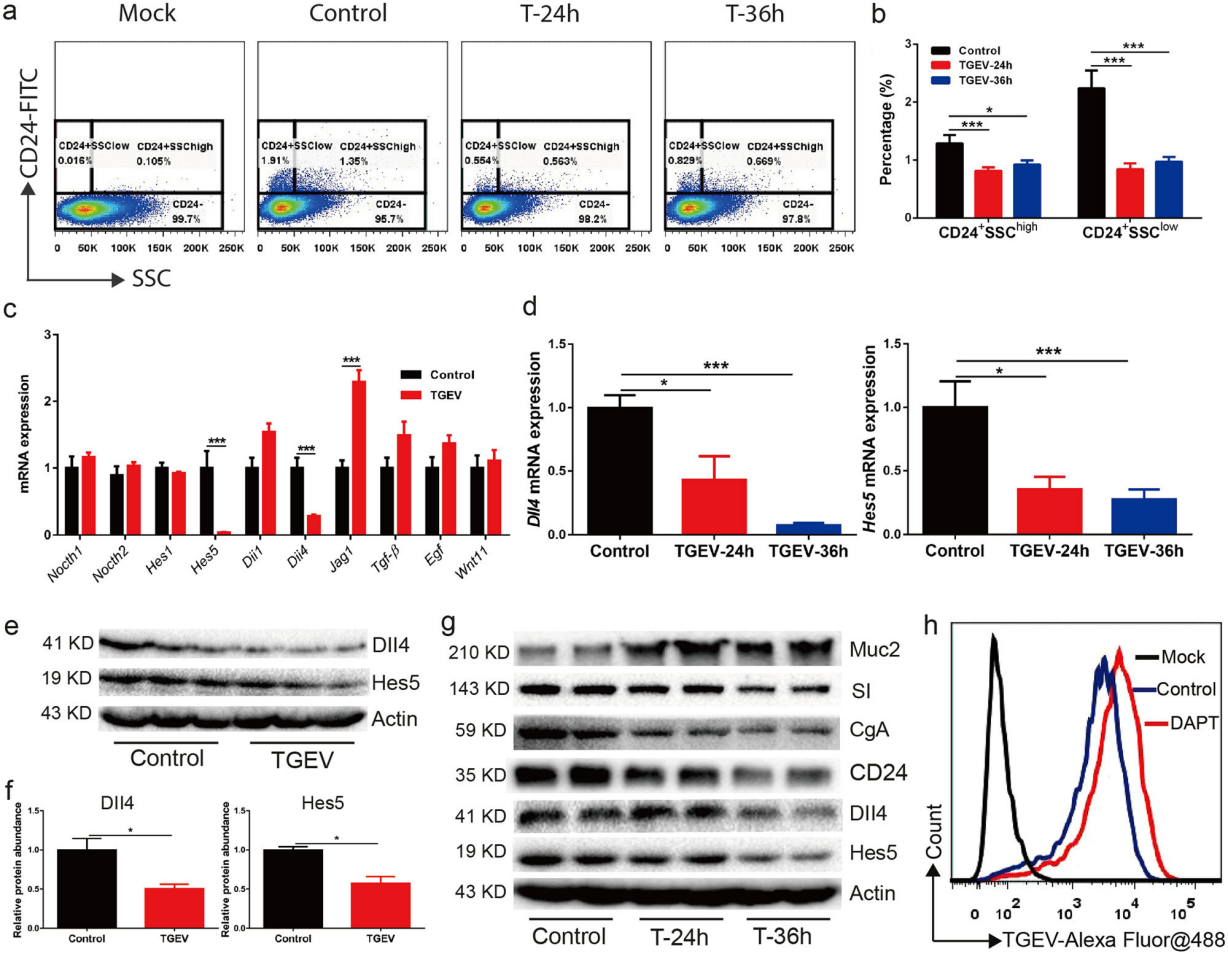
TGEV infection results in CD24^+^SSC^high^ cells (Paneth cells) loss and Notch signaling inactivation. **a** Representative FACS of CD24 in TGEV-infected IPEC-J2 cells. **b** Quantification of CD24^+^SSC^high^ (Paneth cells) and CD24^+^SSC^low^ (enteroendocrine cells) number in TGEV-infected IPEC-J2 cells. **c** Jejunum from TGEV-infected piglets were analyzed by quantitative PCR (qPCR) for makers of ISCs niche signaling (Notch, Wnt, and EGF signaling). **d** Representative Notch factors mRNA change in TGEV-infected IPEC-J2 cells. **e** Western blot for Notch effector Hes5 and Notch ligand DII4 in jejunum from TGEV-infected piglets. **f** Quantitation of bands to demonstrate the protein level of DII4 and Hes5. **g** Representative Notch factors (DII4 and Hes5) and intestinal epithelial cells markers Muc2, SI, CgA, and CD24 were tested by western blot in TGEV-infected IPEC-J2 cells. **h** TGEV content was tested by FACS in 24 h post TGEV-infected IPEC-J2 cells, which were pre-treated by DAPT for 12 h before TGEV infection, and DAPT continued addition during TGEV infection period.

**Fig. 4:**
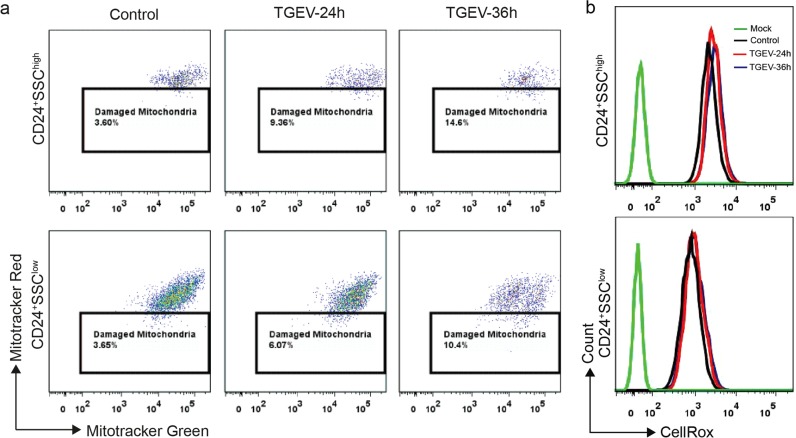
TGEV infection induced mitochondria damage and ROS generation in CD24^+^SSC^high^ cells and CD24^+^SSC^low^ cells. IPEC-J2 cells were infected by TGEV for indicated time points. **a** FACS for cell damaged mitochondria in CD24^+^SSC^high^ cells and CD24^+^SSC^low^cells. **b** FACS for cellular total ROS level in CD24^+^SSC^high^ cells and CD24^+^SSC^low^cells.

To uncover the potential mechanisms for Lgr5 ISCs loss and the impact of TGEV infection on the niche signals for Lgr5 ISCs fate decision, we took advantage of TGEV-infected IPEC-J2 cells model. FACS for CD24 demonstrated that TGEV infection resulted in CD24 cell loss in TGEV-infected IPEC-J2 cells (Supplementary Fig. S3a), and IF staining results also revealed that TGEV infection decreased the expression of CD24 in TGEV-infected jejunum (Supplementary Fig. S3b, c). Subsequently, we found TGEV infection caused Paneth cell (CD24^+^SSC^high^ cells) and enteroendocrine cell (CD24^+^SSC^low^ cells) loss (Supplementary Fig S3d; Fig. 3a, b), as was observed in TGEV-infected jejunum (Fig. 2e–h). We next examined apoptotic cell death and cell proliferation to determine potential mechanisms for Paneth cells loss with TGEV infection. Either early cell apoptosis or late cell apoptosis was robustly induced by TGEV in CD24^+^SSC^high^ cells (Supplementary Fig. S4a, b). Meanwhile, similar results were detected in CD24^+^SSC^low^ cells (Supplementary Fig. S4c, d), but no changes in cell apoptosis were found in CD24^−^cells (Supplementary Fig. S4e, f). Cell apoptosis mainly occurred in CD24^+^ cells. Moreover, combining with FACS analysis of Paneth cells proliferation with Ki67 staining, TGEV infection inhibited the proliferation of Paneth cell (CD24^+^SSC^high^ cells) and enteroendocrine cell (CD24^+^SSC^low^ cells) in TGEV-infected IPEC-J2 cells (Supplementary Fig. S4g, h). Subsequently, we detected the mitochondrial function and ROS generation in TGEV-infected IPEC-J2 cells, which largely contribute to cell apoptosis and barrier functions of intestinal epithelial cells. As expected, TGEV infection significantly induced mitochondria damage and ROS production not only in Paneth cells (CD24^+^SSC^high^ cells) but also in enteroendocrine (CD24^+^SSC^low^ cells) (Fig. 4a, b).

Since Paneth cells provide various niche factors to support ISCs self-renewal and differentiation. Paneth cells loss seriously affects the number and function of ISCs through reducing niche factor generation. To reveal the effect of TGEV on niche factor production, we first detected the mRNA expression of the representative niche factor markers in TGEV-infected jejunum (Fig. 3c) and IPEC-J2 cells (Fig. 3d). These results showed that TGEV infection significantly inhibited Notch ligand *Dii4* and Notch effector *Hes5* mRNA expression. For Wnt (*Wnt11*) and BMP (*Tgf-β)* signaling, no significant changes in mRNA level were observed in TGEV-infected jejunum or IPEC-J2 cells (Fig. 3c, d). Then DII4 and Hes5 protein expression was quantified in TGEV-infected jejunum and IPEC-J2 cells by using WB (Fig. 3e–g). Infection by TGEV disrupted the Notch signaling for Lgr5 ISCs self-renewal and differentiation via down-regulating DII4 and Hes5 protein expression both in in vivo (Fig. 3e, f) and in vitro (Fig. 3g). In addition, TGEV infection decreased SI, CgA, CD24 protein expression (Fig. 3g). Alternatively, goblet cells (Muc2) were up-regulated in TGEV-infected IPEC-J2 cells (Fig. 3g), with similar effect on goblet cells was detected in TGEV-infected jejunum (Fig. 2c, i, j). Subsequently, we inhibited Notch signaling in IPEC-J2 cells by using *N*-S-phenyl-glycine-t-butyl ester (DAPT), a γ-secretase inhibitor, which turns proliferative cells in intestinal crypts and adenomas into goblet cells. As expected, DAPT rapidly induced goblet cells differentiation at the cost of absorptive cells in IPEC-J2 cells (data not shown). Surprisingly, Notch signaling inhibition strongly promoted TGEV infection and replication in IPEC-J2 cells (Fig. 3h). Therefore, these results demonstrate that TGEV infection alters Lgr5 ISCs fate via inducing Paneth cells loss and disrupting Notch signaling factors. Moreover, goblet cells differentiation enhances TGEV infection and replication.

### Paneth cells (CD24^+^SSC^High^) are the initial invasion cells of TGEV

**Fig. 5:**
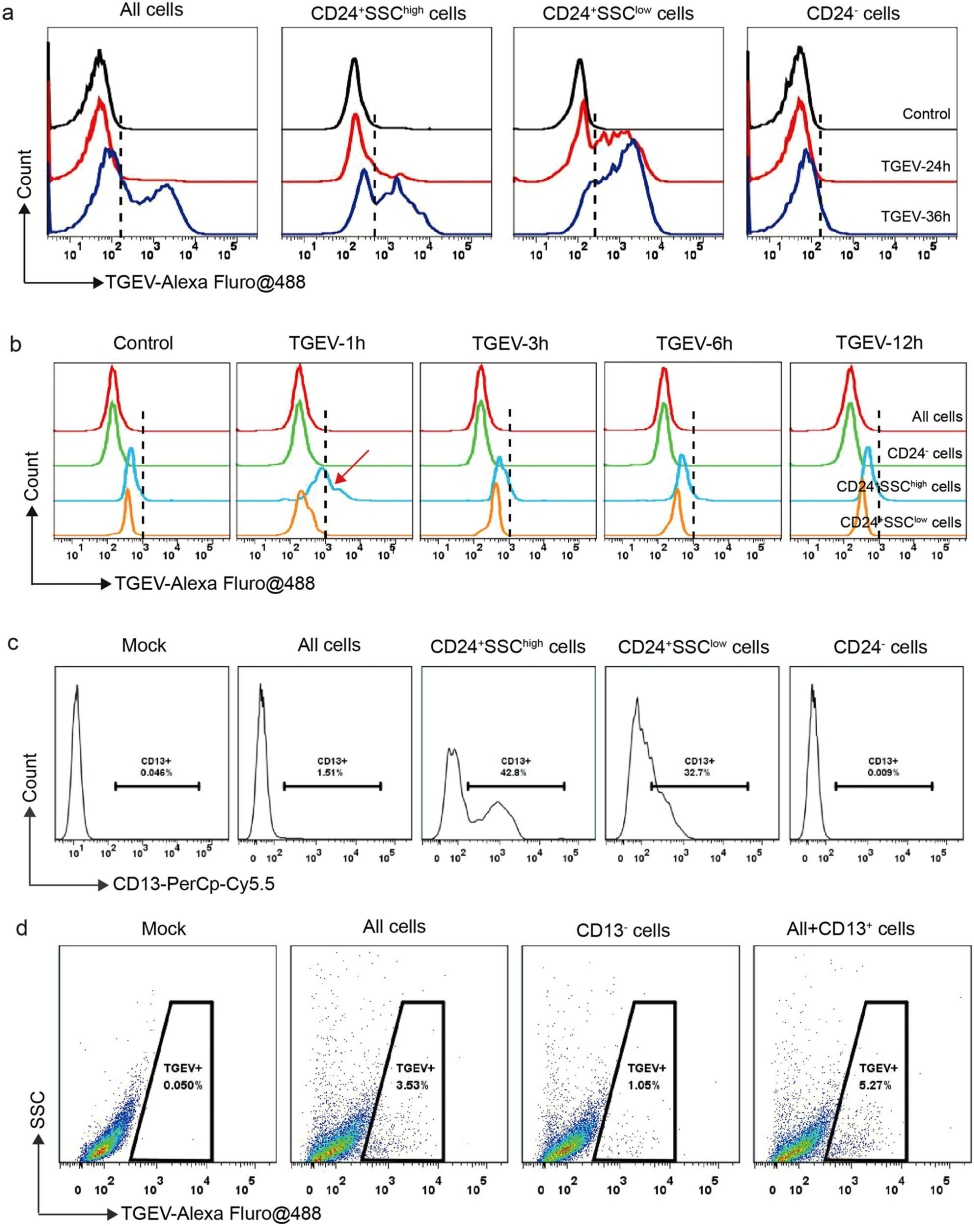
TGEV initial invades CD24^+^SSC^high^ cells (Paneth Cells), which highly express TGEV invasion receptor APN (CD13). IPEC-J2 cells were infected with TGEV for the indicated time points. (**a**, **b**) Staining with TGEV antibody, the percentage of TGEV-infected cells were determined by using FACS. (**c**) FACS for CD13 (TGEV receptor APN) in difference cell types. (**d**) FACS for TGEV content in different cells types after 24 h of TGEV infection.

As noted earlier, TGEV infection distinctly affects the number and function of Paneth cells. To uncover the specific mechanism, we detected the infection proportion and replication of TGEV in IPEC-J2 cells. Of interest, TGEV was mostly detected in CD24^+^ cells, which mainly include CD24^+^SSC^high^ cells (Paneth cells) and CD24^+^SSC^low^ cells (enteroendocrine). In CD24^+^SSC^low^ cells the effect was most pronounced, with the percentage of TGEV-positive cells nearly 10-fold as many as compared to other cell types in 24 h post TGEV-infected IPEC-J2 cells. However, a few cells were infected by TGEV in CD24^−^ cells, which are mostly absorptive enterocytes (Fig. 5a). Although the difference of the infection proportion gradually narrowed in 36 h post infection, CD24^+^SSC^low^ cells still contained more intracellular TGEV. Notably, less TGEV was detected in 36 h post infection Paneth cells, which is opposite with the result of 24 h post infection. These data suggest that TGEV selectively targets cell types for infection and replication. We followed those observations by examining TGEV infection rate over shorter time periods in several cell types. Surprisingly, in contrast to results from 24 and 36 h incubation, after only 1 h TGEV infection was higher in CD24^+^SSC^high^ cells than CD24^+^SSC^low^ or CD24^+^SSC^−^. About 20% of CD24^+^SSC^high^ cells carried TGEV, but only 2% cells were infected with TGEV in other cell types, such as all, CD24^−^ and CD24^+^SSC^low^ IPEC-J2 cells (Fig. 5b). It suggests that TGEV selectively targets to CD24^+^SSC^high^ cells for initial invasion.

### Aminopeptidase N (APN, CD13) receptor is required for TGEV invasion to Paneth Cells

Similar to other coronaviruses (CoVs), TGEV utilizes APN (CD13) as its receptor for cell invasion (Supplementary Fig. S5b). Since TGEV mainly targets Paneth cells for initial invasion, we detected TGEV receptor APN expression in different cell types (Fig. 5c). FACS results showed only about 1.5% cells were CD13 positive in IPEC-J2 cells. However, 42.8% CD24^+^SSC^high^ cells and 32.7% CD24^+^SSC^low^ cells expressed CD13. Notably, almost no CD13 was expressed in other IPEC-J2 cells (CD24^−^ cells) (Fig. 5c). Subsequently, removing CD13^+^ cells from IPEC-J2 cells by using FACS markedly decreased TGEV infection by 60%. As expected, additional supplementation of CD13^+^ cells in IPEC-J2 cells significantly promoted TGEV infection (Fig. 5d). Removing CD13^+^ cells didn’t affect the number of CD24^+^SSC^high^ and CD24^+^SSC^low^ cells in CD13^+^-removed IPEC-J2 cells, even upon TGEV infection (Supplementary Fig. S5a) and rescued Lgr5 ISCs loss, which was induced by TGEV (Supplementary Fig. S5b). In brief, TGEV initially invades Paneth cells through APN (CD13) receptor.

Subsequently, we found that inhibition of TGEV infection by *APN* gene knockout in IPEC-J2 cells rescues the fate of Lgr5 ISCs (Supplementary Fig. S6). This event directly inhibited TGEV infection and replication in APN-KO IPEC-J2 cells (Supplementary Fig. S6b). Comparable to normal IPEC-J2 cells, *APN*-KO IPEC-J2 cells displayed inhibition on cell apoptosis, which was induced by TGEV in Lgr5 ISCs, CD24^+^SSC^high^, CD24^+^SSC^low^ and CD24^−^cells (Supplementary Fig. S6d; Supplementary Fig. S7b–d). Hence, there were no significant decreases in the number of Lgr5 ISCs, CD24^+^SSC^high^ and CD24^+^SSC^low^ cells, even with TGEV infection (Supplementary Fig. S6c and Fig. S7a).

### TGEV non-structural protein 10 and 16 (NSP10 and NSP16) mediates cells proliferation and Lgr5 ISCs fate decision

**Fig. 6:**
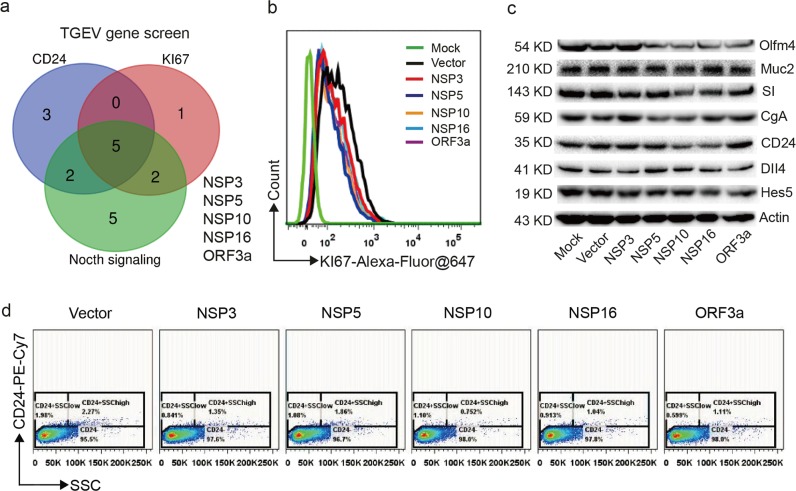
TGEV-encoded NSP10 and NSP16 mediate cell proliferation and Lgr5 ISCs fate. **a** Cell proliferation (KI67), CD24 cells number and Notch signaling factors DII4 and Hes5 were analyzed in TGEV gene stable cell lines. Venn diagram depicts the numbers of decreased cell proliferation and CD24 cells number and down-regulated Notch signaling identified by using FACS and western blot. **b** FACS for KI67 in TGEV NSPs stable cell lines. **c** Notch factor and intestinal epithelium markers Muc2, SI, CgA, and CD24 were tested by western blot in TGEV NSPs stable cell lines. **d** FACS for CD24 in TGEV NSPs stable cell lines.

Naturally, viruses encoded proteins, which determine their epidemiological characteristic and pathogenesis, there is no exception for TGEV. To reveal which TGEV-encoded protein mediates Lgr5 ISCs fate decision. 23 TGEV encode genes (*NSP1-NSP16, ORF3a, ORF3b, M, N, E, S* and *ORF7*) were cloned and constructed within stable cell lines in IPEC-J2 cells. Cell proliferation (KI67 staining), CD24 cell number and Notch signaling (DII4 and Hes5 protein level) were tested in these stable cell lines. FACS for KI67 staining showed that there are 8 TGEV-encoded proteins that inhibit cell proliferation (Supplementary Fig. S8a), and 9 proteins that cause CD24 cells loss (Supplementary Fig. S8c). Moreover, there were 10 TGEV-encode proteins which down-regulated Notch signaling (Supplementary Fig. S8b). As represented via a Venn diagram, five TGEV encode proteins (NSP3, NSP5, NSP10, NSP16, and ORF3a) simultaneously affect cell proliferation, CD24^+^ cell number and Notch signaling (DII4 and Hes5 protein expression) (Fig. 6a). Among these viral proteins, NSP16 was the most significant inhibitor of cell proliferation and Notch signaling (Fig. 6b, c), and strongly decreased CD24^+^SSC^high^ and CD24^+^SSC^low^ cell number (Fig. 6d). In addition, NSP16 down-regulated SI, CD24 and CgA protein level, but significantly up-regulated Muc2 protein expression (Fig. 6b). Moreover, NSP16 decreases Olfm4 expression in NSP16 stable cell lines (Fig. 6b). Similar results were observed in TGEV-infected jejunum (Fig. 2c–j). Therefore, we focused on NSP16, which mediated cell proliferation and Lgr5 ISCs fate decision. Of note, NSP10 induces similar phenomenon to NSP16 in IPEC-J2 cells (Fig. 6; Supplementary Fig. S8).

### TGEV-encoded NSP10/NSP16 protein complex not only interact with DII4 but also alter DII4 promoter activity

**Fig. 7:**
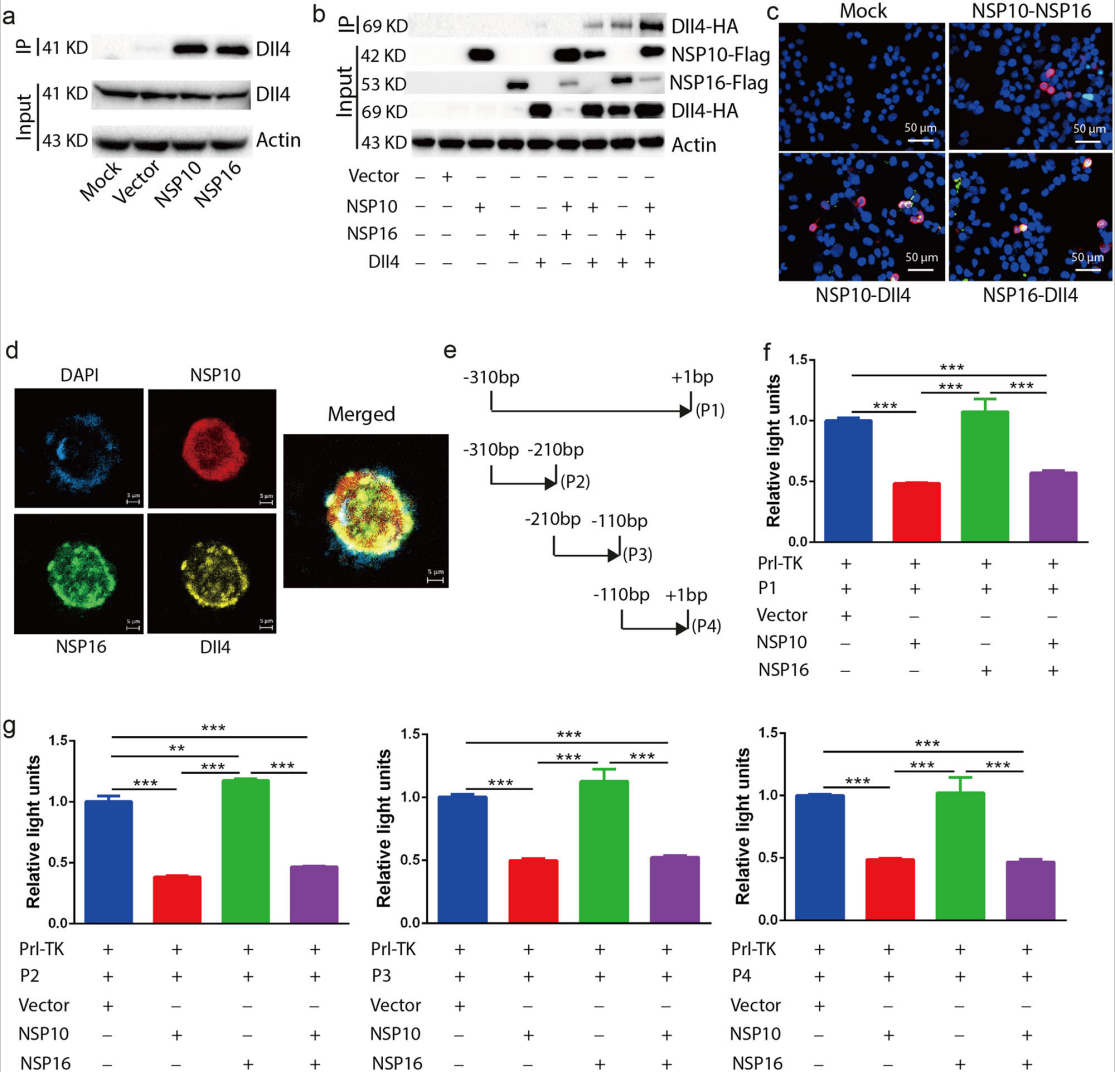
TGEV-encoded NSP10/NSP16 protein complex not only interact with DII4 but also alter DII4 promoter activity. **a** IPEC-J2 cells were stable transfected with empty vector, pLVX-AcGFP1-Flag-NSP10 and pLVX-AcGFP1-Flag-NSP16. Cell lysates were immunoprecipitated with anti-Flag mAbs followed by immunoblot of DII4 mAbs to assess the interaction between NSP10, NSP16 and DII4 protein. **b** HEK-293T cells were transfected with empty vector, pLVX-DsRed-Monomer-Flag-NSP10, pLVX-AcGFP1-Flag-NSP16 and/or pLVX-mVenus-HA-DII4 plasmids. Cell lysates were co-immunoprecipitated with anti-Flag® M2 antibody followed by immunoblot of DII4 using anti-HA mAbs to assess the interaction between NSP10, NSP16 and DII4 protein. **c** HEK-293T cells were co-transfected with pLVX- AcGFP1-Flag-NSP10, pLVX-AcGFP1-Flag-NSP16 and pLVX-mVenus-HA-DII4. A representative result of Duolink PLA. Pink fluorescence indicates PLA signal (Scale bars, 50 μm). **d** HEK-293T cells were co-transfected with pLVX-DsRed-Monomer-Flag-NSP10, pLVX-AcGFP1-Flag-NSP16 and pLVX-mVenus-HA-DII4. Then, the cells were fixed at 36 h post transfection to detect the co-localization of NSP10, NSP16, and DII4 protein (Scale bars, 5 μm). **e** Potential promoter regions and the information of three different fragments of potential promoter regions are listed. The start site of exon 1 is designated as +1 bp. **f** The relative luciferase activities of P1 (−310 bp) following NSP10 and/or NSP16 co-transfection. **g**) The relative luciferase activities of P2 (−310 bp to −210 bp), P3 (−210 bp to −110 bp) and P2 (−110 bp to +1 bp).

Normally, NSP16 activity is regulated by NSP10, and forms a protein complex with NSP10 to inhibit the translation and/or stability of host proteins in other CoVs, such as SARS-CoV and MERS-CoV. Moreover, TGEV and TGEV-encoded NSP10 and NSP16 distinctly inhibit Notch signaling factors (DII4 and Hes5). Therefore, we investigated whether TGEV-encoded NSP10 and/or NSP16 alter Notch signaling via interacting with these factors (DII4 and/or Hes5). To explore this point, we performed IP experiments in NSP10 and NSP16 stable transfected IPEC-J2 cell lines, and CO-IP experiments in NSP10 and NSP16 transiently transfected HEK239T cells. Anti-Flag immunoprecipitation followed by anti-DII4 and anti-HA western blotting showed specific binding of NSP10 and NSP16 to DII4 (Fig. 7a, b), but no interactions were detected between NSP10 and Hes5 as well as NSP16 and Hes5. To further test their interactions between NSP10, NSP16 and DII4, we utilized a Duolink proximity ligation assay (PLA), which can demonstrate protein-protein interactions in situ by eliciting a fluorescent signal. PLA signals were detected in DII4-HA co-transfection with NSP10-Flag and/or NSP16-Flag cells, revealing the interaction of these proteins (Fig. 7c). We also tested for the interaction between TGEV-encoded NSP10 and NSP16 by using PLA. Indeed, NSP10 was observed to bind to NSP16 (Fig. 7c). To test whether the co-localization of NSP10, NSP16 and DII4 occurs, HEK239T cells were co-transfected with pLVX-DsRed-Monomer-Flag-NSP10, pLVX-AcGFP1-Flag-NSP16, pLVX-mVenus-HA-DII4 and fixed at 36 h post transfection. The results showed that NSP16 not only co-localized with NSP10, but also co-localized with DII4 (Fig. 7d), which further confirmed the interaction between TGEV-encoded NSP10, NSP16 and DII4. These results suggest that TGEV-encoded NSP10 and NSP16 form a complex in the host to alter DII4 protein level.

To reveal the potential mechanism of NSP10 and NSP16 in reducing DII4 protein level, we cloned a fragment spanning −310 bp to +1 bp (P1) of the *DII4* predicted promoter into the pGL3-Basic vector (Fig. 7e). HEK293T cells were co-transfected with P1, Prl-TK (Renilla luciferase control reporter vectors), Vector, NSP10 and/or NSP16. We found that NSP10 robustly down-regulates DII4 promoter (P1) activity. However, NSP16 did not alter the transcriptional activity of *DII4* promoter (Fig. 7f). Subsequently, we divided *DII4* promoter (P1) into three sections (Fig. 7e) and detected the promoter activity of these fragments by using dual-luciferase reporter system. NSP10 was observed to inhibit the promoter activity of three different *DII4* promoter fragments by about 50–60% (Fig. 7g). Although NSP16 slightly enhanced the *DII4* promoter (P2) activity by about 10%, NSP10 still inhibited the *DII4* promoter (P2) activity even in the presence of NSP16 (Fig. 7g).

## Discussion

**Fig. 8:**
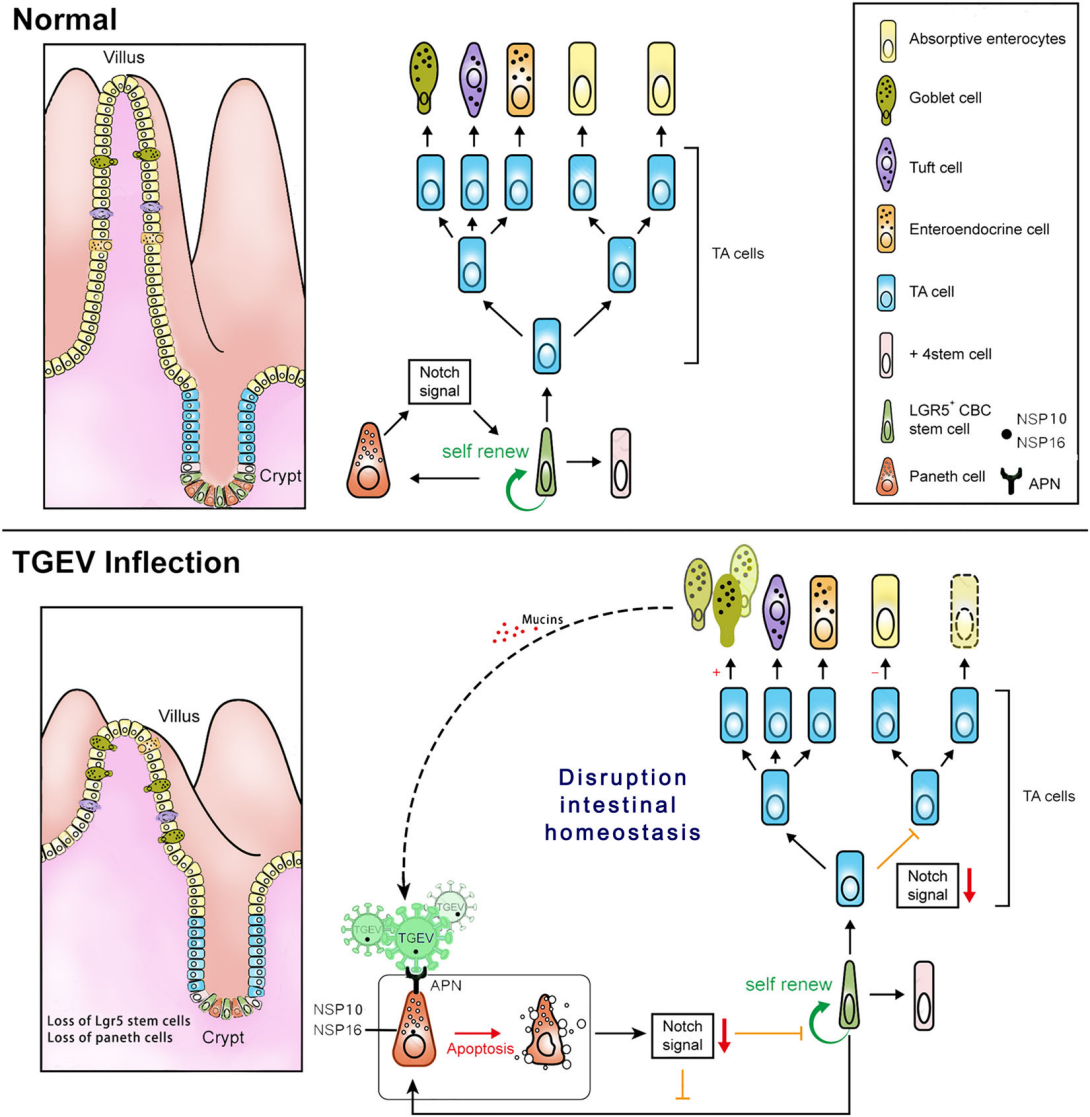
Model of proposed mechanism of TGEV-mediated disruption of intestinal homeostasis. In the case of normal condition(the upper panel), Lgr5 ISCs continuously generate rapidly proliferating TA cells, which differentiate into the various functional cells on the villi (enterocytes, tuft cells, goblet cells and enteroendocrine cells) to replace the intestinal epithelial cells being lost via anoikis at the tip of villi. Paneth cells are unusual in that they intercalate with Lgr5 ISCs and provide niche factors (such as Notch signaling) for Lgr5 ISCs fate decision, which is essential for intestinal epithelial cells renewal and intestinal homeostasis maintenance. Upon TGEV infection (the lower panel), TGEV targets Paneth cells for initial invasion via APN receptor and robustly induces cell apoptosis in Paneth cells, which causes a dramatic loss of Paneth cells number and niche factor (Notch signaling) for Lgr5 ISCs self-renewal and differentiation. Notch signaling, a switch between secretory lineages and absorptive lineages differentiation, plays a vital function in intestinal cells fate decision. Notch signaling inhibition induced goblet cells differentiation at the cost of absorptive enterocytes. Interestingly, mucus secreted by goblet cells contains sialic acid, which promotes TGEV infection. Thus, as time goes on, the more TGEV invades intestinal epithelial cells, the more severe damage occurs in vivo. A vicious circle is gradually forms in TGEV-infected jejunum. This flawlessly explains why sharp villus atrophy occurs in TGEV-infected jejunum. The whole process is mediated by TGEV-encoded NSP10 and NSP16, which interactions regulate Lgr5 ISCs fate and intestinal homeostasis.

It is now well established that intestinal crypt cells respond to damage induced by high-dose irradiation or chemicals by activation of reserve stem cells^5,25–27^. Here, we reveal intestinal crypt cells exhibit a novel response to a diarrheal virus (Fig. 8). In this study we found that TGEV infection results in villous atrophy within 48 h and inhibits intestinal epithelium renewal by halting the self-renewal and differentiation of Lgr5 ISCs. As the epithelium of the intestine is the fastest renewing tissue, sustained by Lgr5 ISCs^28^, once Lgr5 ISCs lose the ability of self-renewal and diferentiation, it will seriously affect intestinal epithelium turnover and perturb intestinal homeostasis. A recent report similarly showed that *Heligmosomoides polygyrus* infection causes Lgr5 ISCs loss through activating IFN-γ generation and induces fetal-like reversion in the intestinal stem-cell niche^4^. Lgr5 ISCs are apoptosis sensitive cells to different types of stresses (such as ROS), so it is easy to be attacked^29^. Previous study demonstrated that TGEV-encoded N protein induced ROS generation, which contributes to cell apoptosis activation via p53 signaling in ST cells^30^. Although TGEV induces mildly ROS production in TGEV-infected IPEC-J2 cells, it robustly promotes ROS generation in Lgr5 ISCs (data not shown), which provides a potential explanation of Lgr5 ISCs loss and why more apoptosis cells were observed in crypt.

Except for cell apoptosis, TGEV-mediated Paneth cells loss also contributes to the developmental fate of Lgr5 ISCs. TGEV directly invades Paneth cells through the APN receptor, and then activates ROS generation, which ultimately induces Paneth cells apoptosis. Paneth cell loss severely affects the niche factors secretion (such as Wnt and Notch factors) needed for Lgr5 ISCs self-renewal and differentiation^14^. Among these niche factors, Notch factor is often linked to developmental patterning^31^, intestinal stem-cell self-renewal and crypt homeostasis^20^. Although a few studies shown that Paneth cells are dispensable for survival, proliferation, and stem-cell activity^32,33^. This because the other cell type severs the same function as paneth cells, such as mesenchymal cells^14,34,35^. Beyond stimulating Lgr5 ISCs cells with niche signals, Paneth cells also provide essential nutrients to ISCs^1^. Therefore, paneth cells are essential for Lgr5 ISCs. In our study TGEV infection significantly down-regulates Notch ligand DII4 and Notch effector Hes5 protein expression both in vitro and in vivo. Inactivation of DII4 causes the loss of stem and progenitor cells^22^. Thus, Paneth cells loss, affecting Notch signaling activation, may be the foundational mechanism of TGEV-mediated inhibition of Lgr5 ISCs self-renewal and differentiation.

Notch signaling is a developmental switch for intestinal secretory cells and absorptive enterocytes^2^, as its suppression leads to a block of differentiation of enterocytes and a dramatic increase in the number of goblet cells^23^. As villus are mainly composed of enterocytes, TGEV infection most likely causes villous atrophy. Indeed, TGEV infection promotes progenitor cells differentiation to goblet cells at the cost of absorptive cells in TGEV-infected jejunum. TGEV infection causes villous atrophy within a short time (generally within 48 h), and the villus-height/crypt-depth ratio rapidly changed from 7:1 to 1:1^36^. Intriguingly, TGEV infection promotes goblet cells differentiation and accompanies an increase of mucins secretion. Contrary to the luminal protection of gastrointestinal tract, mucins have the ability to promote TGEV infection. As mucins are rich in sialic acids^37^, they are interaction partners for TGEV and thus may help to penetrate the mucus layer to gain access to APN on the surface of the intestinal epithelial cells^38^. Thus, over time, the more TGEV enters into intestinal epithelium, the more severe damage occurs in TGEV-infected jejunum. A circle promoting infection is then gradually formed within 24 h of TGEV infection. This is, at least in part, reason for villous sharp shortness and high mortality of TGEV infection. Thus, the mucins are a “double-edged sword” that needs to be balanced gently. Base on these results, it is not difficult to find that the disruption of TGEV mainly occur in crypt, and villous atrophy is the ultimate result of crypt damage. This “from bottom to top” finding of TGEV pathogenesis to intestine is quite novel to the traditional “top down” understanding of TGEV infection that villus damage occurs first and damage to crypt next. Together, our data suggests that the Notch pathway might represent the cell developmental “switch” of Lgr5 ISCs and Paneth cells in the case of TGEV infection. Therefore, Notch signaling may be an attractive therapeutic target for TGEV infection. The fact that Notch signaling controls key steps of differentiation in most and has become therapeutic targets for many diseases^39^.

Subsequently, we found that TGEV-encoded NSP16 significantly attenuates Notch signaling (DII4 and Hes5) activation. NSP16, a 2′-*O*-methyltransferase (2′-*O*-MTase) plays a crucial role for 2′-*O*-methylation of viral mRNA in capping of viral RNA, which permits viral infection with reduced host recognition^40,41^. The absence of 2′-*O*-methylation or NSP16 mutant activates a more robust type I IFN response that ablates viral infection and replication^41,42^. Thus, capping of viral RNA by NSP16 is an effective and successful strategy to disrupt host immune recognition. For TGEV, its encoded NSP16 not only helps itself to evade host recognition, which promotes TGEV infection and replication, but also inhibits Notch signaling via inhibiting the DII4 promoter activity, and ultimately disrupts intestinal homeostasis. Generally, COVs NSP16 activity is strictly dependent on its interaction with NSP10^42,43^, this is no exception for TGEV. TGEV-encoded NSP10 interaction with NSP16 to prevent virus infection by cell innate immunity mechanisms. Inhibition of NSP10/NSP16 MTase activities by A MTase specific inhibitor AdoHcy significantly inhibits TGEV infection and replication (data not shown). Additionally, NSP10, NSP16 and their complex can interact with DII4, which normally binds to Notch receptors. This interaction may disturb Notch signaling. Notably, NSP10 not only binds to DII4, but also down-regulates DII4 promoter activity, which provides a good explanation of TGEV and its NSP10 and/or NSP16-mediated *DII4* mRNA and protein decrease. Therefore, NSP10/NSP16 complex is likely another novel therapeutic target for TGEV infection.

In summary, our findings highlight a link between intestinal crypt cells response and virus infection that regulates Paneth cells function and Lgr5 ISCs fate. TGEV infection causes loss of Notch signaling, which inhibits Lgr5 ISCs self-renewal and differentiation and causes villous atrophy. This phenomenon can also be regarded as an explanation of TGEV pathogenicity, and possibly represents similar pathways used by other diarrheal virus (such as rotavirus) and bacteria, to disrupt intestinal homeostasis. Given its central role in TGEV infection, Notch signaling is an attractive therapeutic target for TGEV infection. Future studies will explore how intestinal crypt regenerates the damaged gut with diarrheal virus infection.

## Materials and methods

### Animals and TGEV infection

A total of 12 7-days-old DLY male piglets were obtained from a swine herd at Sichuan Agricultural University and artificially fed with milk for three days. After that, all piglets were randomly divided into two groups, the TGEV-infected group (TGEV) and the control group (Control) in this study (*n* = 6), and then piglets were orally inoculated with either 0 or 1 × 10^9^ PFU TGEV (TCID50 = 10^−7^/100 μl) according to the previous assignment (Control vs. TGEV infected). Each experimental group of piglets was housed in a separate room in a high-security isolation facility. Piglets that developed significant diarrhea and lived two days after infection were used in the experiment. The experimental procedures used in this study were approved by the Animal Care Advisory Committee of Sichuan Agricultural University.

### Histological analysis, immunofluorescence and in fluorescence situ hybridization

Intestinal tissue was collected and fixed overnight in 4% PFA in PBS and embedded in paraffin. In all, 5-μm sections were stained by HE and PAS for histological analysis and goblet cells staining. Sample were embedded in OCT and 8-μm sections were performed for immunofluorescence staining by using the following primary antibodies: TUNEL (Roche, 11684817910), anti-Ki67 (BD; 550609), anti-CD24 (Thermo Fisher; MA5-11828), anti-Muc2 (Santa Cruz; sc-515032), anti-CgA (Immunostar; 20086). All primary antibodies were used as 1:100 dilutions. Goat anti-mouse Alexa fluor®488 (Abcam, ab150113) and goat anti-mouse Alexa fluor®647 (ab150115) secondary antibodies were used at 1:500–1:1000 dilutions.

For fluorescence in situ hybridizations (FISH), tissues of jejunum were fixed overnight in 4% PFA, paraffin embedded, and sectioned at 10 μm. The probe sequences targeting *Lgr5*, *Olfm4* and *Lys (Lysome)* was: *Lgr5* probe (5′-FAM-GACGACAGGCGGTTGGACGATAGGT-FAM-3′), *Olfm4* probe (5′-FAM-CACTGACACCTCGCCACCATTCCA-FAM-3′) and *Lys* probe (5′-CY3-GCACCGATCATAGACCTTGGCCTGTA-3′). The protocols used for in vitro transcription and in situ hybridization were previously described^44^. All images were acquired and processed with Zeiss Axio-Imager Z1 with apotome or Leica SP5 confocal microscope.

### Cell culture and viral infection

IPEC-J2 cells were obtained from ATCC and cultured in DMEM/F12 supplemented with 10% FBS, 100IU/ml of penicillin, 100 mg/ml of streptomycin, 5 mg/ml hEGF and 10 nM HEPES, at 37 °C in a 5% CO2 atmosphere incubator. TGEV was provided by Prof. Zhiwen Xu. Confluent (70%) IPEC-J2 cells were inoculated with TGEV at MOI of 1 for 1 h at 37 °C. The unattached virus were removed and the cells were washed one time with PBS. Subsequently, fresh growth medium was added.

### TGEV gene screen and stable cell lines constructs

The TGEV genes were constructed by RT-PCR amplification from the genomic RNA of TGEV strain WH-1 and cloned into lentiviral vector pLVX-AcGFP1-N1. All plasmids were confirmed by sequencing and transfected into HEK293T packaging cells with pLP/VSVG and psPAX2 two plasmids packing system. Stably transfected IPEC-J2 cells were selected with 2.5 μg/ml puromycin selection for 2 weeks. Selected cells were tested for TGEV genes mRNA expression by reverse transcription-PCR (RT-PCR).

### Flow cytometry and cell sorting

Flow cytometry (FACS) experiments were performed with the following antibody: Alexa Fluor®647-Lgr5 (BD; 562912), FITC-CD24 (BD; 555427), PE-Cy7TM-CD24 (BD; 561646), PerCP-CyTM 5.5-CD13 (BD; 561361). FACS measurements were performed using a BD-FACS service device and analyzed with FlowJo software (FlowJo LLC). MFI is defined as the difference in the signal intensity between an unstained control and a stained sample. For sorting, a FACS Aria SORP device (Becton Dickinson) was used.

### Quantification of reactive oxygen species and damaged mitochondria

For the detection of ROS, 5 μM CellRox Deep Red reagent (Life Technologies; C10422) was incubated with IPEC-J2 cells culture for 30 min at 37 °C. Dysfunctional mitochondria were monitored by fluorescence levels upon staining with 100 nM MitoTracker Green FM (Life Technologies; M7514) and 100 nM MitoTracker Red CMXRos (Life Technologies; M7512) for 30 min at 37 °C. Subsequently, cells were washed three times with PBS and analyzed by flow cytometry.

### Cell apoptosis and proliferation

Apoptotic cell death was detected by FITC-, Alexa Fluor®647- conjugated Annexin V with propidium iodide (PI) staining assay (Biolegend; 640914 and 610912) after the manufacturer’s protocols. Briefly, IPEC-J2 cells were harvested, then 10^6^ cells were resuspended in 100 μL 1 × binding buffer followed by incubation with 2 μL /10^6^ cells Annex V per test for 20 min on ice. Subsequently, 400 μL 1 × binding buffer and 1 μL PI (1 mg/ml) was added to the sample and immediately analysis by FACS. Cell proliferation was determined by using FACS. IPEC-J2 cells infected with TGEV were harvested and fixed with 4% formalin in PBS for 15 min at room temperature. Fixed cells were permeabilized with 0.05% Triton X-100 in PBS at room temperature for 15 min. For intracellular Ki67(BD; 550609) staining, cells were incubated for 2 h with Ki67 antibody (0.5 μg/10^6^ cells), followed by secondary antibody staining with 1:1000 dilution of goat anti-mouse Alexa fluor®488 (Abcam; ab150113) for additional 1 h.

### qPCR and western blotting

Total RNA was extracted from samples using TRIzol reagent (Invitrogen) according to the manufacturer’s protocol and cDNA synthesised using the prime script^TM^ RT reagent kit with gDNA eraser (Takara, RR047A). Relative gene expression was calculated with the ∆∆C_T_ method, normalizing the results to the value for the *Gapdh* gene. Protein extraction and western blotting were performed as described previously, using primary antibodies against Olfm4 (Abcam; ab85046), SI (Santa Cruz; sc-27603), CD24 (Thermofisher; MA5-11828), Muc2 (Santa Cruz; sc-515032), CgA (ImmunoStar; 20086), Hes5 (Santa Cruz; sc-293445), DII4 (Abcam; ab7280) and β-Actin (Santa Cruz; 47778). The dilution of primary antibodies was 1:1000. The second goat anti-rabbit and goat anti-mouse antibodyies conjugated to HPR (Santa Cruz; sc-2030 and sc-2031) was used (1:3000).

### CRISPR-Cas9 knockout of pANPEP

The porcine *ANP* gene (gene ID: 397520) was disrupted in IPEC-J2 cells by using the CRISPR-Cas9 technique. In brief, two guide RNAs (gRNAs) targeting the first exon of the *ANP* gene, GGTAGGCGGTACCGGTTCCA (ANP-KO-1) and GCGTTGTGGGTAGGCGGTAC (ANP-KO-2), and complementary oligonucleotides were designed. The annealed gRNA duplexes were cloned into the lentiCRISPRv2 vector (Addgene; 52961) by using the BsmBI restriction site. The gRNA-expressing vectors were transfected into HEK239T cells, and cell culture supernatants containing viral particles were harvested and used for infection of IPEC-J2 cells. IPEC-J2 cells were subsequently subjected to puromycin selection for 2 weeks. Selected clones were tested for *ANP* mRNA expression by reverse transcription-PCR (RT-PCR). A representative clone from each of the two CRISPR constructs was used in the experiments described here.

### Immunoprecipitation (IP) and co-immunoprcipitation (CO-IP)

NSP10 and NSP16 stable IPEC-J2 cells were harvested and lysed in lysis buffer. 200 μg of cell lysate protein was incubated with 2.5 μg anti-Flag® M2 antibody (CST; 14793) and 20 μl of protein

A magnetic beads (CST; 73778) overnight at 4 °C. Samples were washed three times with lysis buffer, resuspended in 2x sample buffer and analyzed with immunoblot analysis using DII4 antibody. For CO-IP experiments, Hek293T cells expressing NSP10-Flag, NSP16-Flag and/or DII4-HA constructs were harvested and lysed in lysis buffer. Samples were then centrifuged to remove precipitated proteins and were incubated with anti-Flag® M2 antibody overnight at 4 °C. Samples were washed three times with lysis buffer, resuspended in 2x sample buffer and analyzed with immunoblot analysis using the indicated antibodies.

### Luciferase reporter assay

HEK293T cells were seed into 48-well plates at 3 × 10^5^ cells/well and co-transfected with different DII4 predicted promoter plasmids, NSP10 and NSP16. All cells were transfected with the Renilla luciferase control plasmid Prl-TK (Promega, E2241). Luciferase activity was measured with a Dual-Luciferase assay kit (Beyotime; RG027) with Glomax 96 microplate luminometer (Promega) in luminometer mode following the manufacturer’s protocol. The raw values of firefly luciferase were normalized to Renilla luciferase.

### Duolink proximity ligation assay

HEK239T cells expressing NSP10-HA, NSP16-Flag or DII4-HA construct were co-cultured on a cover slip. Cells were fixed with 4% PFA for 15 min at room temperature, blocked with 0.1 gelation-PBS, and stained with 1:250 rabbit anti-Flag and 1:250 mouse anti-HA antibodies for 1 h at room temperature. Cells were then stained with 1:000 donkey anti-rabbit plus and donkey anti-mouse minus second antibodies. After washing with 0.1% gelatin-PBS, proximity ligation assay (PLA) was done according to manufacturer’s protocol (Sigma).

### Statistical analysis

Each of the experiments described here was performed in at least three independent biological replicates. Statistical analysis was performed by using Graph Pad Prism software. All results were unpaired two-tailed Student’s *T* test and/or one-way analysis of variance (ANOVA). *P* ≤ 0.05 were considered to be statistically significant (*p < 0.05, **p < 0.01, ***p < 0.001).

## Supplementary information

Supplemental figure legends

Supplemental Figure S1

Supplemental Figure S2

Supplemental Figure S3

Supplemental Figure S4

Supplemental Figure S5

Supplemental Figure S6

Supplemental Figure S7

Supplemental Figure S8
