# Supplementary figures and images for: Transmissible gastroenteritis virus targets Paneth cells to inhibit the self-renewal and differentiation of Lgr5 intestinal stem cells via Notch signaling
Source: Cell Death Dis. 2020 Jan 20;11(1):40. doi: 10.1038/s41419-020-2233-6 (PMC6971083; doi:10.1038/s41419-020-2233-6)

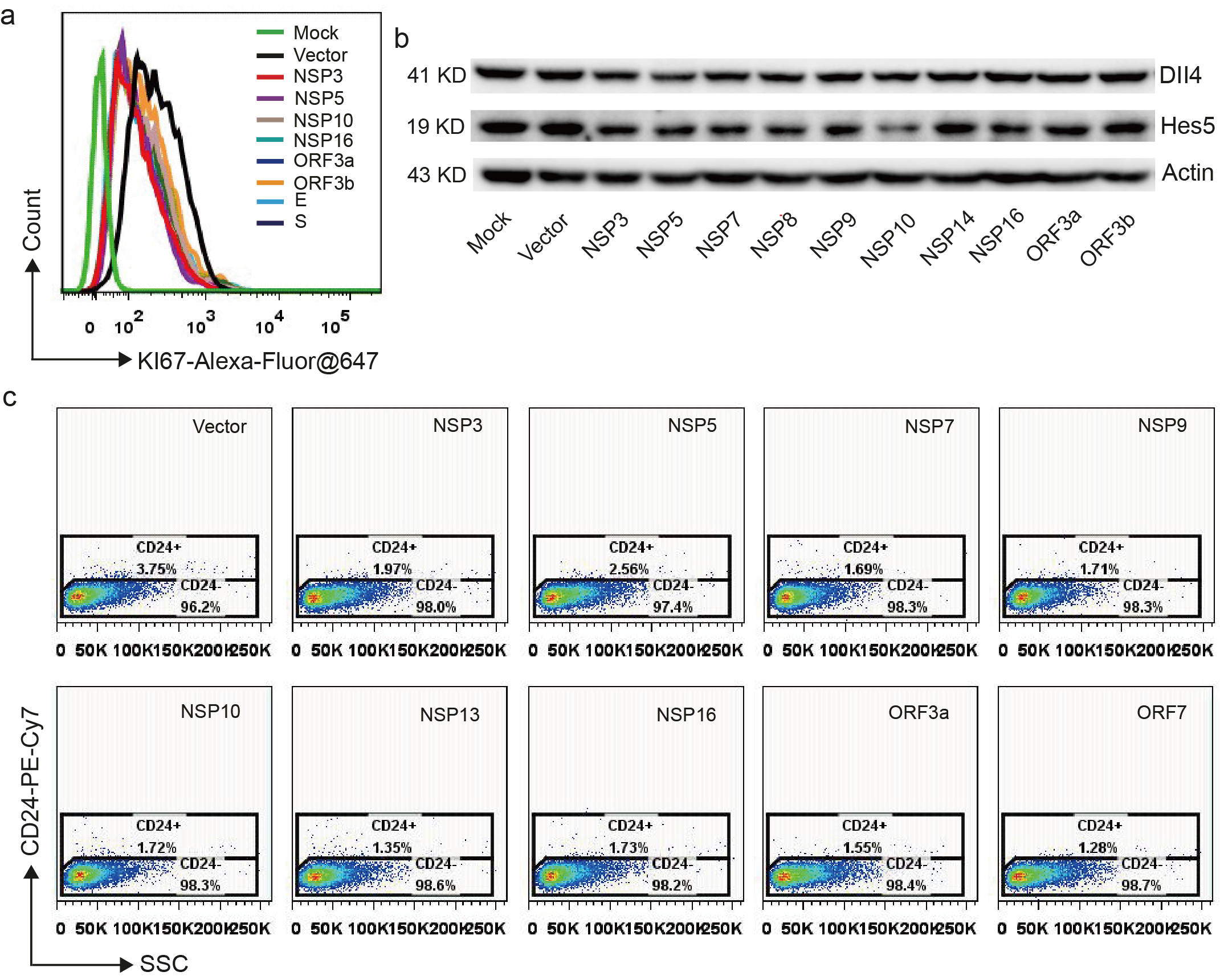

Supplement: Supplementary file 1 — Supplemental figure legends [file 41419_2020_2233_MOESM1_ESM.png]

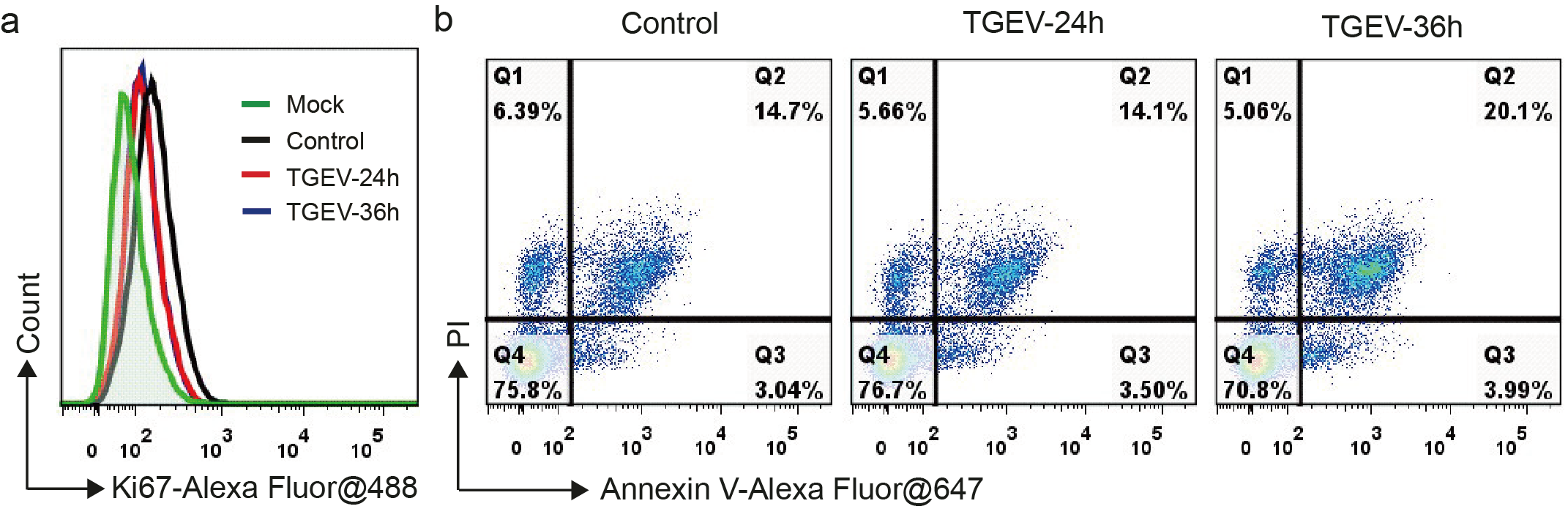

Supplement: Supplementary file 3 — Supplemental Figure S2 [file 41419_2020_2233_MOESM3_ESM.png]

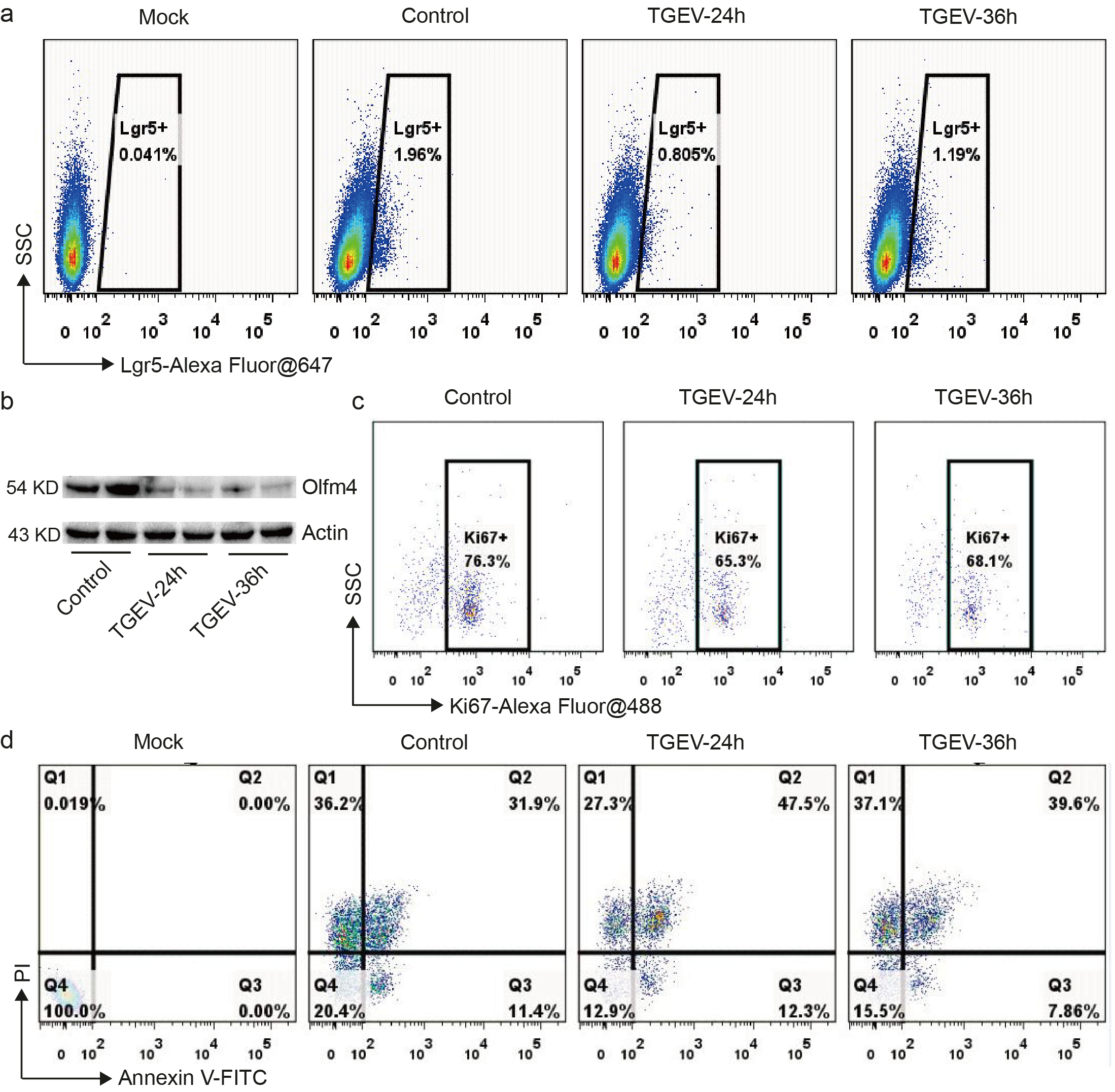

Supplement: Supplementary file 4 — Supplemental Figure S3 [file 41419_2020_2233_MOESM4_ESM.png]

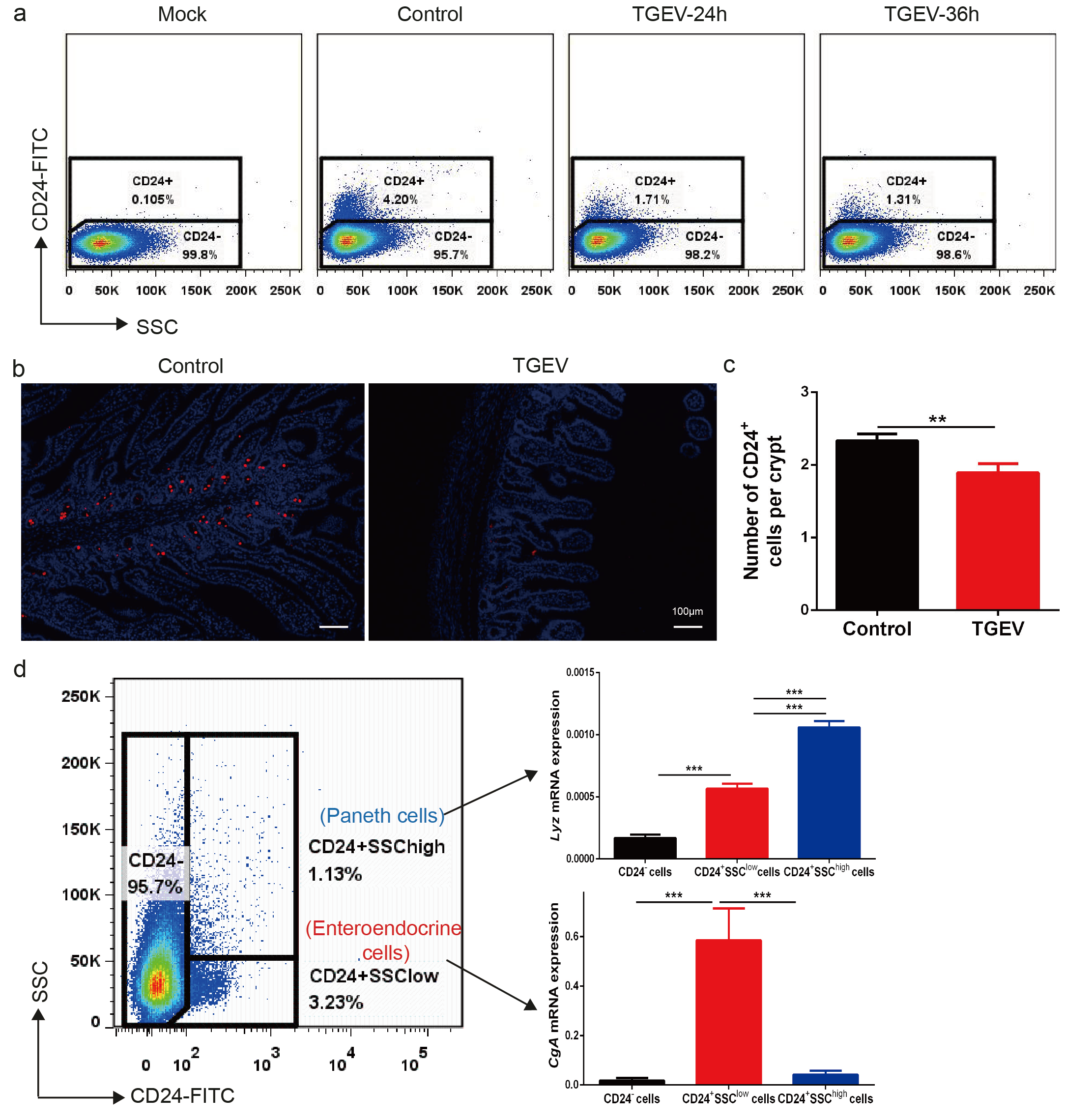

Supplement: Supplementary file 5 — Supplemental Figure S4 [file 41419_2020_2233_MOESM5_ESM.png]

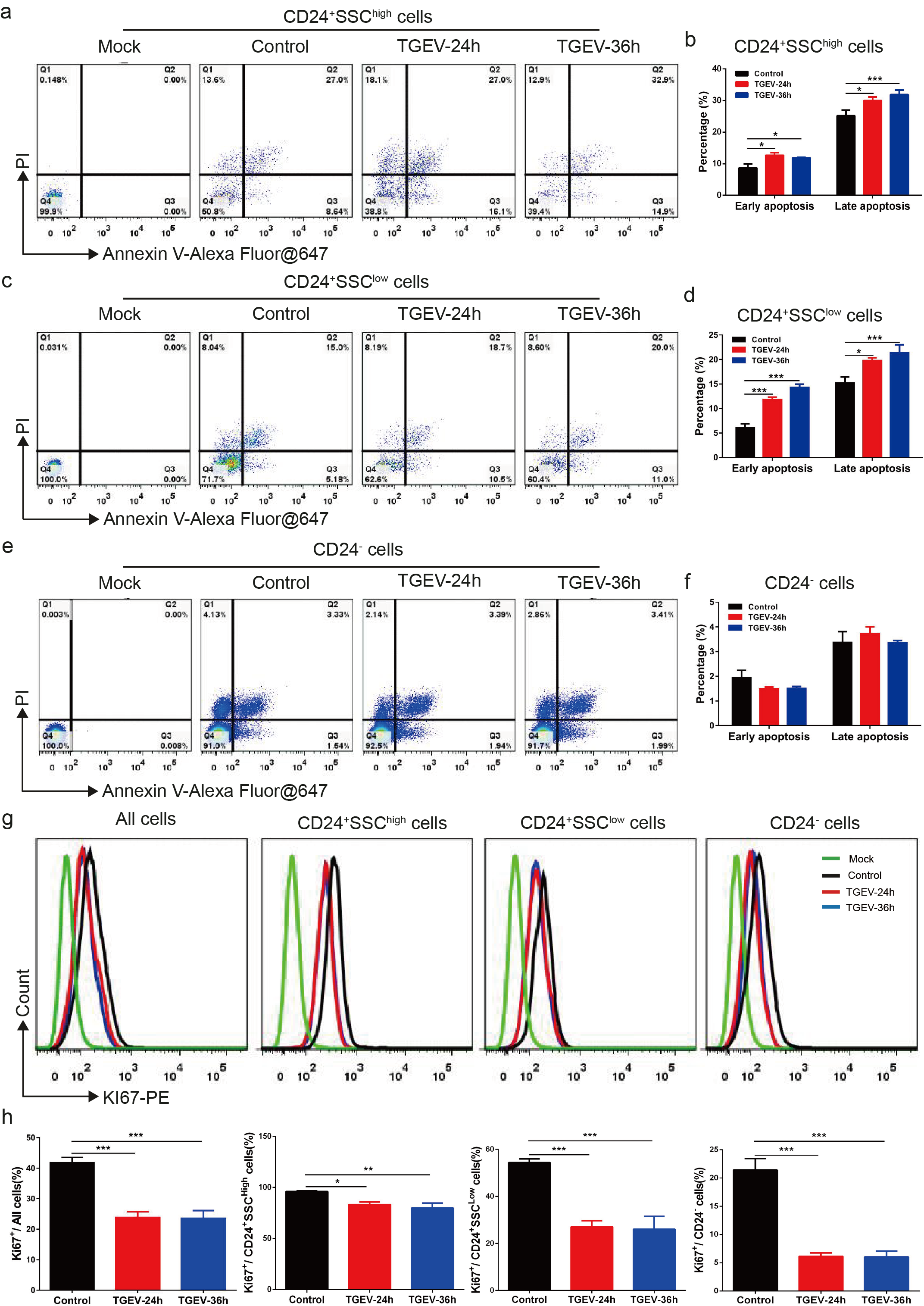

Supplement: Supplementary file 6 — Supplemental Figure S5 [file 41419_2020_2233_MOESM6_ESM.png]

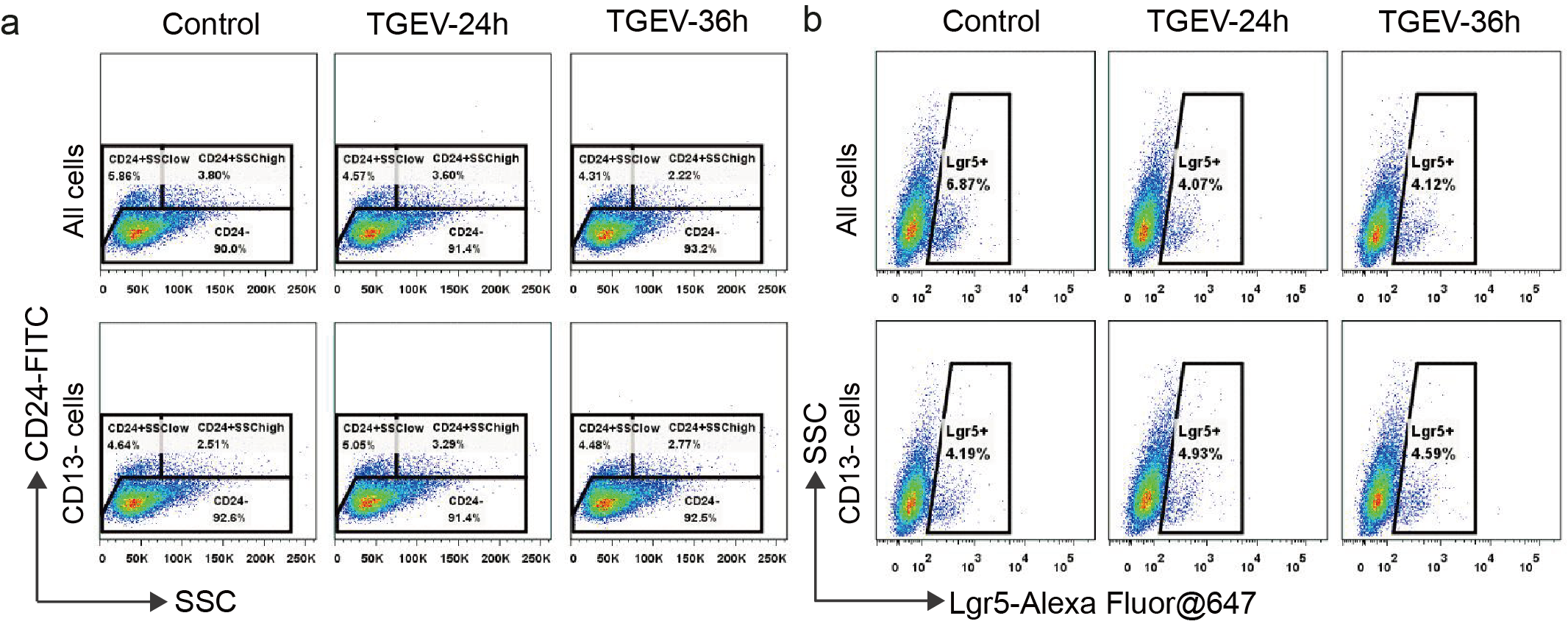

Supplement: Supplementary file 7 — Supplemental Figure S6 [file 41419_2020_2233_MOESM7_ESM.png]

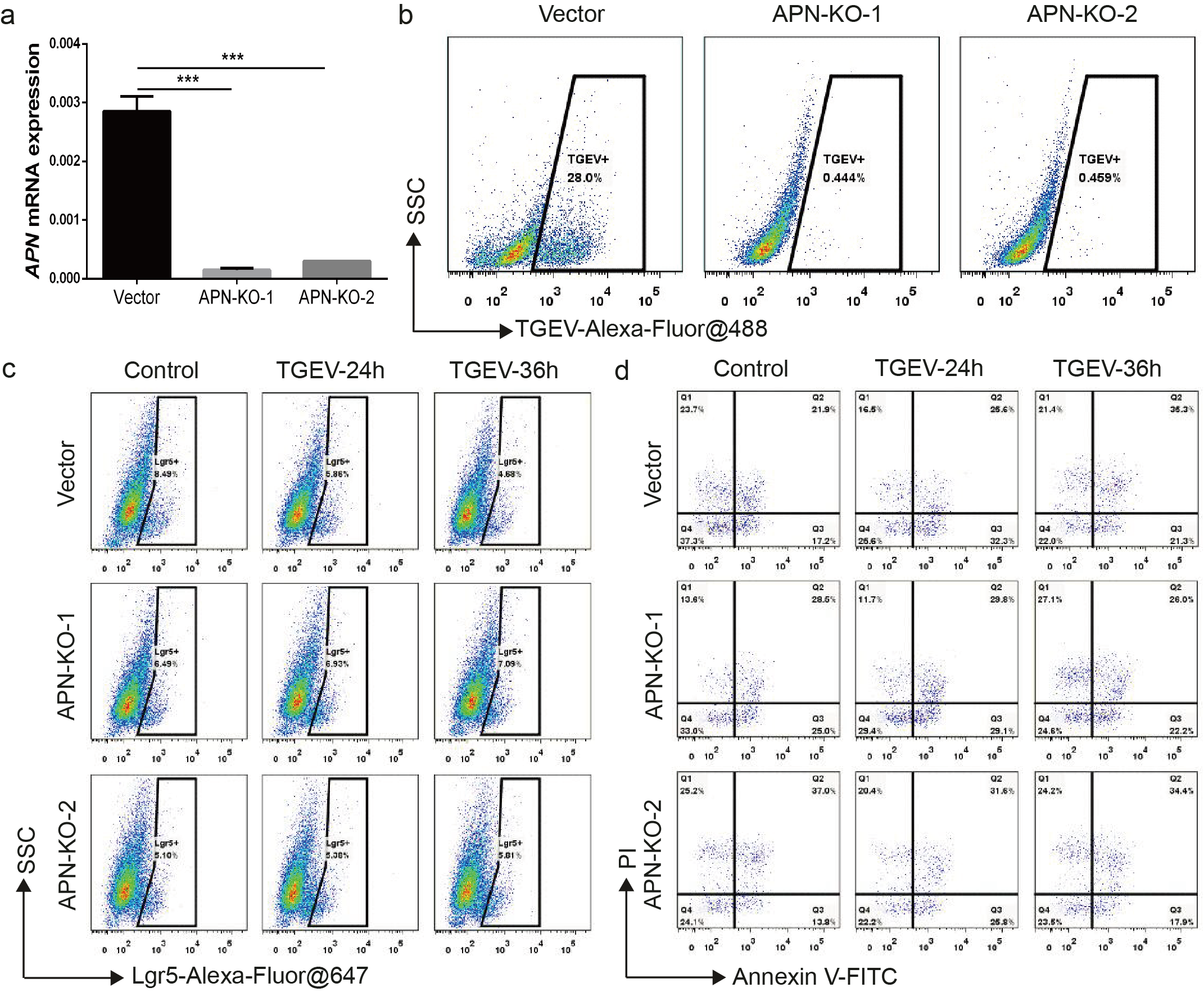

Supplement: Supplementary file 8 — Supplemental Figure S7 [file 41419_2020_2233_MOESM8_ESM.png]

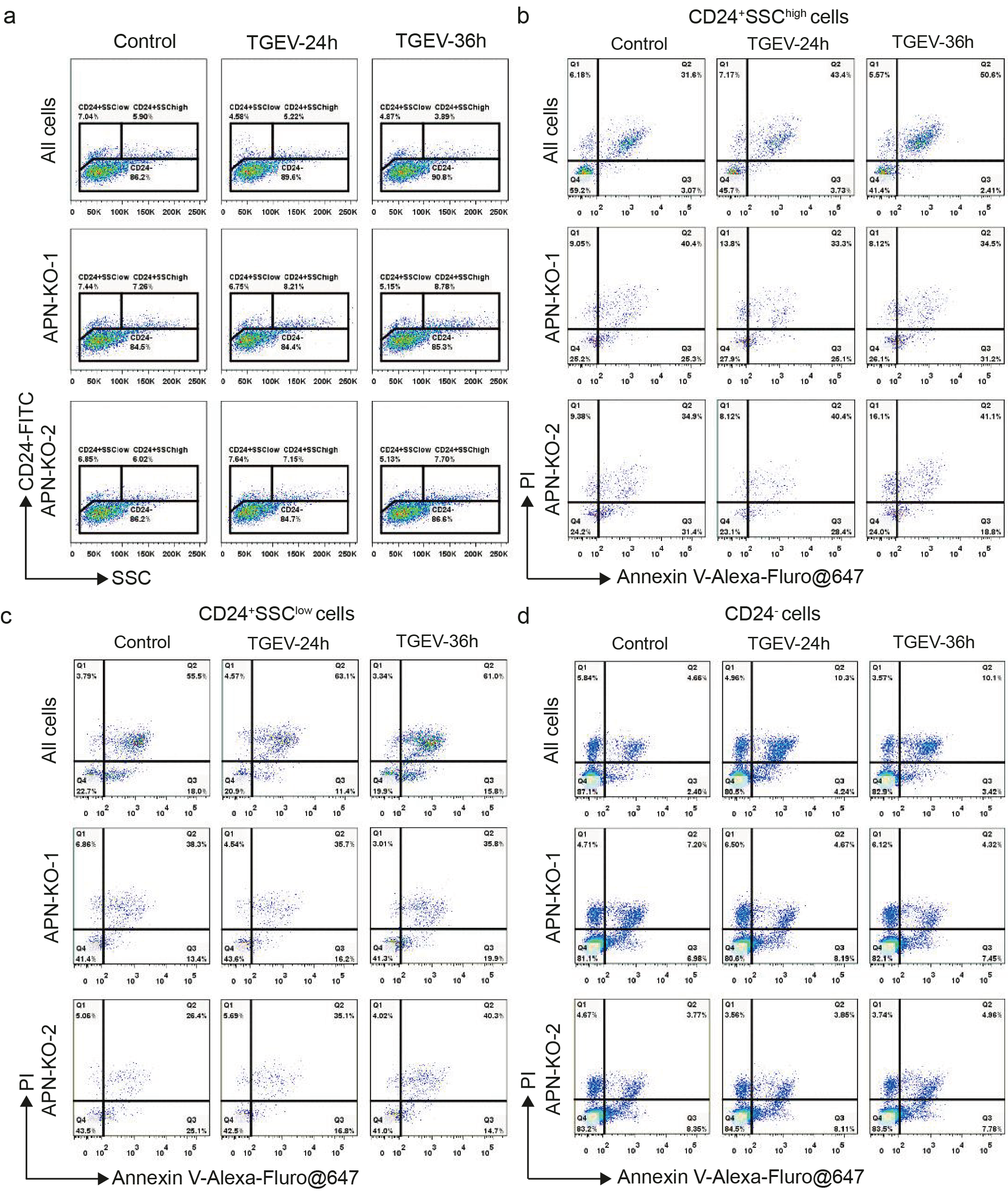

Supplement: Supplementary file 9 — Supplemental Figure S8 [file 41419_2020_2233_MOESM9_ESM.png]
