# Supplementary material for: Transmissible gastroenteritis virus targets Paneth cells to inhibit the self-renewal and differentiation of Lgr5 intestinal stem cells via Notch signaling
Source: Cell Death Dis. 2020 Jan 20;11(1):40. doi: 10.1038/s41419-020-2233-6 (PMC6971083; doi:10.1038/s41419-020-2233-6)
Supplement: Supplementary file 2 — Supplemental Figure S1 [file 41419_2020_2233_MOESM2_ESM.docx]

**Supplementary information**

**Supplemental figure legends**

**Supplementary Fig. S1 TGEV infection inhibits cell proliferation and merely induces cell apoptosis in IPEC-J2 cells.**

(a) FACS for cell proliferation in TGEV infected IPEC-J2 cells by using KI67 staining. (b) FACS for cell apoptosis in TGEV infected IPEC-J2 cells.

**Supplementary Fig. S2 TGEV infection results in Lgr5 and Olfm4 ISCs loss**

(a) FACS for Lgr5 in TGEV infected IPEC-J2 cells for indicated time points. (b) Western blot for Olfm4 in TGEV infected IPEC-J2 cells. (c) Cell proliferation of Lgr5 ISCs were measured by FACS in TGEV infected IPEC-J2 cells. (d) Cell apoptosis of Lgr5 ISCs were determined by FACS in TGEV infected IPEC-J2 cells.

**Supplementary Fig. S3 TGEV infection causes CD24^+^ cells loss in vitro and in vivo**

(a) FACS for CD24 in TGEV infected IPEC-J2 cells for indicated time points. (b and c) Jejunal cross-section stained with CD24 (Scale bars, 100μm) and quantification of the CD24 positive cells per crypt (n=150). (d) Sorting CD24^+^SSC^high^, CD24^+^SSC^low^ and CD24^-^ cells from IPEC-J2 cells by FACS, then *Lyz* and *CgA* mRNA expression were tested by qPCR.

**Supplementary Fig. S4 TGEV infection not only increases cell apoptosis but also suppresses cell proliferation.**

(a) Representative FACS for cell apoptosis in CD24^+^SSC^high^ (Paneth cells). (b) Quantification of cell apoptosis rate in CD24^+^SSC^high^ cells. (c and d) Representative FACS for cell apoptosis in CD24^+^SSC^low^ cells. (e and f) Representative FACS for cell apoptosis in CD24^-^ cells. (g) Representative FACS for cell proliferation in IPEC-J2 cells (All cells), CD24^+^SSC^high^ cells, CD24^+^SSC^low^ cells and CD24^-^ cells. (h) Quantification of cell proliferation rate in these different cell types.

**Supplementary Fig. S5 Removing CD13^+^ cells form IPEC-J2 cells rescues TGEV-induced Paneth cells and Lgr5 ISCs loss**

(a) FACS for CD24 in different cells types for the indicated time points. (b) FACS for Lgr5 ISCs in different TGEV infected cells types.

**Supplementary Fig. S6 Inhibition TGEV infection by *APN* gene knockout rescues Lgr5 ISCs fate**

(a) *APN* mRNA expression in APN-deleted (APN-KO) IPEC-J2 cells. (b) Post 36 h TGEV infection, the percentage of TGEV-containing cells was tested by FACS in TGEV infected IPEC-J2 cells. (c) FACS for Lgr5 ISCs in TGEV infected normal and APN-KO IPEC-J2 cells. (d) FACS for cells apoptosis in Lgr5 ISCs in normal and APN-KO IPEC-J2 cells.

**Supplementary Fig. S7 *APN* gene knockout decreases cell apoptosis in CD24^+^SSC^high^ cells and CD24^+^SSC^low^ cells**

(a) The percentage of CD24^+^SSC^high^ cells and CD24^+^SSC^low^ cells in normal and APN-KO IPEC-J2 cells. (b, c and d) FACS for cells apoptosis in CD24^+^SSC^high^ cells, CD24^+^SSC^low^ cells and CD24^-^ cells in normal and APN-KO IPEC-J2 cells, which infected with TGEV for 24 h or 36h.

**Supplementary Fig. S8 TGEV encoded non-structural proteins mediate CD24 cells number loss, cell proliferation inhibition and Notch signaling inactivation**

(a) FACS for KI67 in TGEV NSPs stable cell lines. (b) Notch factors and intestinal epithelial cells markers Muc2, SI, CgA and CD24 were tested by western blot in TGEV NSPs stable cell lines. (c) FACS for CD24 in TGEV NSPs stable cell lines.
